# Study protocol of an economic evaluation of an extended implementation strategy for the treatment of low back pain in general practice: a cluster randomised controlled trial

**DOI:** 10.1186/s13012-014-0140-x

**Published:** 2014-10-08

**Authors:** Cathrine Elgaard Jensen, Allan Riis, Kjeld Møller Pedersen, Martin Bach Jensen, Karin Dam Petersen

**Affiliations:** Danish Center for Healthcare Improvements, Faculty of Social Sciences and Faculty of Health Sciences, Aalborg University, Aalborg, Denmark; Research Unit for General Practice in the North Denmark Region, Aalborg, Denmark; Department of Clinical Medicine, Aalborg University, Aalborg, Denmark; Centre of Health Economics Research, Faculty of Business and Social Sciences, University of Southern Denmark, Odense, Denmark

**Keywords:** Study protocol, Implementation strategies, Economic evaluation, Cost-effectiveness, Cost-utility, Guideline adherence, Compliance, Randomised clinical trial, Low back pain

## Abstract

**Background:**

In Denmark, guidelines on low back pain management are currently being implemented; in association with this, a clinical trial is conducted. A health economic evaluation is carried out alongside the clinical trial to assess the cost-effectiveness of an extended implementation strategy to increase the general practitioners’ adherence to the guidelines. In addition to usual dissemination, the extended implementation strategy is composed of visits from a guideline facilitator, stratification tools, and feedback on guideline adherence. The aim of this paper is to provide the considerations on the design of the health economic evaluation.

**Methods/design:**

The economic evaluation is carried out alongside a cluster randomised controlled trial consisting of 60 general practices in the North Denmark Region. An expected 1,200 patients between the age of 18 and 65 years with a low back pain diagnosis will be enrolled. The economic evaluation comprises both a cost-effectiveness analyses and a cost-utility analysis. Effectiveness measures include referral to secondary care, health-related quality of life measured by EQ-5D-5L, and disability measured by the Roland Morris disability questionnaire. Cost measures include all relevant additional costs of the extended implementation strategy compared to usual implementation. The economic evaluation will be performed from both a societal perspective and a health sector perspective with a 12-month time horizon.

**Discussion:**

It is expected that the extended implementation strategy will reduce the number of patients referred to secondary care. It is hypothesised that the additional upfront cost of extended implementation will be counterbalanced by improvements in clinical practice and patient-related outcomes, thereby rendering the extended implementation strategy cost-effective.

**Trial registration:**

ClinicalTrials.gov: NCT01699256

**Electronic supplementary material:**

The online version of this article (doi:10.1186/s13012-014-0140-x) contains supplementary material, which is available to authorized users.

## Background

Low back pain is one of the most common musculoskeletal disorders worldwide, and it has been estimated that low back pain affects between 80% and 85% of the general population at least once in their lifetime [1]. Consequently, low back pain constitutes a substantial personal and socioeconomic burden. In Denmark, it has been estimated that the 2011 socioeconomic cost due to back pain amounts to € 1.6 billion [2].

According to a review by Koes et al. [3], there are international consensus on recommendations for management of low back pain; these include, among others, advice to avoid bed rest, use of analgesics, recognition of psychosocial factors, and reduction of secondary care consumption. Furthermore, Koes et al. show that these recommendations have been consistent over the past decade. On these grounds, Koes et al. conclude that future efforts should concentrate on the implementation of guidelines for low back pain management, as systematic implementation is rare, and therefore, patients may receive suboptimal treatment [3]. This is in agreement with a paper by Krismer and van Tulder [4]. In this paper, it is emphasised that health professionals should follow the issued guidelines to ensure the beneficial treatment effects and reduce the personal and socioeconomic burden of low back pain [4].

In Denmark, the new guidelines on low back pain management in general practice are largely in agreement with the guidelines evaluated in Koes et al. [3,5]. These guidelines have the potential to improve quality of care and to save costs by reducing the number of patients referred to secondary care. Utilising secondary care resources optimally is essential, as it has been estimated that, currently, 78% of the annual cost of treatment for back pain in Denmark is associated with secondary care [2].

In this study, it is hypothesised that greater adherence to the guidelines may improve patient-related outcomes and, possibly, decrease utilisation of secondary care. The cost-effectiveness of extending the implementation strategy is, however, unknown.

In association with the publication of the Danish guidelines, a clinical trial is conducted. Alongside the clinical trial, an economic evaluation is conducted to assess the cost-effectiveness of an extended implementation strategy aimed to increase adherence to the guidelines, compared to the usual implementation strategy in Denmark. Further information on, among others, development of the extended implementation strategy and recruitment to the clinical trial are provided elsewhere [6].

The aim of the present paper is to provide the considerations on the design of the economic evaluation carried out alongside the clinical trial.

## Methods/design

The clinical trial is a cluster randomised controlled trial where 60 general practices are enrolled from a sample frame of 191 general practices in the North Denmark Region. It was initially planned to include a total of 2,700 patients through 100 general practices. All practices, except practices used for pilot testing (*n* = 2), were invited to participate in the clinical trial. Due to fewer general practices that wished to participate, the number of practices has been revised to 60 enrolling approximately 1,200 patients. The clinical trial is due to be completed July 2015.

This economic evaluation comprises cost-effectiveness analyses and a cost-utility analysis where the primary outcomes are incremental cost-effectiveness ratios (ICERs). The economic evaluation will be performed from both a societal perspective and a health sector perspective with a 12-month time horizon equal to the follow-up period of the clinical trial.

### Study population

Patients consulting their general practitioner for a new episode of low back pain diagnosed with ICPC-2 diagnosis codes L02, L03, L84, or L86 are eligible for inclusion [7]. Moreover, the patients must understand Danish and be aged 18 to 65 years. The general practitioner invites the patient to participate in the clinical trial and hands out the first of four questionnaires. In this questionnaire, the patient is encouraged to give informed consent. Pregnant women and patients presenting with red flags (signs of serious pathology, e.g. cancer, osteoporosis, fracture) are excluded.

### Trial design

#### Usual implementation strategy

In the control group, the general practitioners are introduced to the clinical guidelines (see the succeeding list) through usual dissemination, consisting of newsletters and information meetings. The general practitioners will manage their patients, including referrals to secondary care, as usual. As an additional feature of usual care, all general practitioners participating in the clinical trial have the opportunity to refer low back pain patients for assessment at the Department of Social Medicine at Aalborg University Hospital.

The main recommendations in the clinical guidelines on management of low back pain in Danish general practice [5,8] are as follows:

- Guidelines for assessment and referral of patients with degenerative disorders of the spine
  1. Course of treatment should include planned consultations 2, 4, and 8 weeks after the initial visit for patients not improving.
  2. The initial consultation should include triage, classifying the patient as having nonspecific low back pain, nerve root pain, or red flags.
  3. Anamnesis should include pain intensity, symptoms, duration of pain, and consumption of analgesics.
  4. The general practitioner is to advise the patient to avoid bed rest and, as far as possible, carry on normal activities.
  5. The general practitioner is to advise the patient on use of analgesics and may refer to supplement primary care, e.g. physical training.
  6. If no satisfactory progress is reached within 8 weeks, the patient should be referred for secondary care.

#### Extended implementation strategy

In addition to usual dissemination, general practitioners allocated to the intervention group will receive further introduction to the guidelines through three interventions: (a) visits from a guideline facilitator. When enrolling in the clinical trial, the general practice receives a visit from a guideline facilitator, who will inform the practitioner about the clinical guidelines and discuss management of low back pain patients. In this clinical trial, five physiotherapists with special certification in low back pain assessment are trained as facilitators. (b) Access to two patient risk-stratification tools that have been added into the general practitioners’ electronic medical records: the subgroups for targeted treatment back screening tool (STarT) and social risk screening (SOS) questions. STarT divides the patients into three groups according to their risk of prolonged symptoms and guide treatment [9]. STarT has been validated in Danish [10]. SOS has been developed for the clinical trial and addresses barriers for recovery including occupational factors, disability compensation or pension claims, and psychosocial factors [6]. Earlier studies have previously indicated that patients involved in, for instance, a compensation claim have poor recovery prognosis, emphasising the importance of identifying these patients as early as possible in order to initiate proper treatment [11,12]. In the clinical trial, the SOS stratification tool is applied to identify patients who are eligible for treatment at the Department of Social Medicine.

The third component of the intervention is (c) feedback on guideline adherence. Throughout their participation in the clinical trial, the general practitioners will have contact with their guideline facilitator and, furthermore, have access to quality reports on their treatment of patients with low back pain compared to other clinics in the North Denmark Region.

### Data collection

When the general practitioner enters one of the ICPC-2 codes into the electronic medical record, a pop-up is activated. The pop-up is activated at each consultation concerning low back pain with the patient and collects data on, among other factors, triage (nonspecific low back pain, nerve root pain, and red flags), duration of pain, symptoms, previous episodes of low back pain, supplementary treatment, and referrals to secondary care. In the intervention group, the two patient risk-stratification tools STarT and SOS are likewise included in the pop-up.

## The economic evaluation

### Resource consumption and estimation of costs

Costs will be calculated in euros (€) for each participant in the clinical trial. A summary of the source of resources and corresponding source of unit costs is provided in Table 1. The economic evaluation is performed as a within-trial analysis, indicating that only costs and effects that accumulate within the trial length are included. Costs are identified and valued as all disease-related direct costs, e.g. contact with primary and secondary health care and patient costs, and indirect costs, which include both absenteeism and presenteeism.

**Table 1:** **Measurements of resources and costs**

**Type of cost**	**Cost for**	**Specification**	**Source of resource**	**Source of unit costs**
Direct cost	Primary care consultations	General practitioner, medical specialist, physiotherapist, and chiropractor	Pop-up and patient in comparison with the Danish National Health Insurance Service Registry	The Danish National Health Insurance Service Registry
	Secondary care consultations	Inpatient, emergency department, and outpatient services	The Danish National Patient Registry	The Danish National Patient Registry
	Diagnostic tests	MRI, CT scan, and X-ray	The Danish National Patient Registry	The Danish National Patient Registry
	Pharmaceutical treatment	Analgesic, NSAIDs, gastric protectors, tramadol, and antidepressants	Patient, Danish Medicines Agency	Danish Medicines Agency
	Transportation	Travel expenses for the patient	Distance between home address and general practitioner	Mileage allowance according to the Danish National Tax Board
	Pop-up	Development	DAK-E	DAK-E
	Usual implementation	Newsletters and informational meetings	Nord-KAP	Nord-KAP
	Extended implementation	Guideline facilitator education and guideline facilitator visits	Nord-KAP, guideline facilitator	Nord-KAP
Indirect cost	Absenteeism	Absence from work	Patient, DREAM	Mean wage according to Statistics Denmark
	Presenteeism	Reduced productivity at work due to low back pain	Patient	Mean wage according to Statistics Denmark

*DAK*-*E* Danish quality unit of general practice, *Nord*-*KAP* quality unit for general practice in the North Denmark Region, *DREAM* the Danish Register of Sickness absence and compensation benefits and social transfer payments.

Primary healthcare utilisation will be based on service fees reimbursed to general practitioners, while secondary healthcare utilisation will be valued by means of the Danish case-mix system. Furthermore, patient costs, such as costs of medication and transportation will be estimated. The human capital approach will be applied to estimate the costs of absenteeism. The impact of estimating these by use of the friction method and of excluding indirect costs will likewise be investigated [13,14]. Patient-reported decrease in productivity due to low back pain is used to estimate presenteeism.

Implementation costs, including costs of pop-up development, education of guideline facilitators, and the ongoing contact between the general practitioners and the guideline facilitators are to be included. To maintain a conservative approach to cost estimation, the implementation costs are not amortised.

### Effectiveness measures

The number of referrals from primary care to secondary care within the first 12 weeks following the initial consultation at the general practice is used as one of the effectiveness measures in the cost-effectiveness analysis. As previously mentioned, consumption of secondary care is a potent cost driver. Hence, increasing the proportion of successful treatment courses in primary care may decrease secondary costs and, subsequently, total costs. Furthermore, decreasing the number of referrals may indicate an increased adherence to the clinical guidelines as well as increased quality of treatment in primary care, rendering the number of referrals as an appropriate effectiveness measure.

#### Patient-related effectiveness measures

The patient-related effectiveness measures are among the most commonly used in low back pain research [15–17] and are as follows: health-related quality of life (HRQoL) and disability. HRQoL is estimated using the EuroQol 5-dimension 5-level (EQ-5D-5L), while disability is evaluated by the use of the Roland Morris disability questionnaire (RDQ; running from 0–23; lower scores indicate a lower degree of disability).

The patient-related effectiveness measures are captured through a questionnaire four times during the trial: at baseline and after 4, 8, and 52 weeks. Furthermore, pain is rated on a numerical rating scale (scale 0–10; a low score indicating less pain), while educational and occupational status, treatment satisfaction, use of medication, presence of comorbidity, and whether the patient has received counselling on activity level are recorded.

#### Health-related quality of life

HRQoL is estimated from EQ-5D-5L. The EQ-5D instrument is used to calculate quality-adjusted life-years (QALYs), which is the most commonly used measure of HRQoL and is recommended in international guidelines [18]. The EQ-5D instrument consists of two parts: the EQ-5D descriptive system and the EQ visual analogue scale (VAS; scale 0–100; a lower score indicates a lower health-related quality of life), respectively. The descriptive system measures the patient’s HRQoL at five levels of severity in five dimensions: mobility, self-care, usual activities, pain/discomfort, and anxiety/depression. This yields 3,125 health states, which are converted to a HRQoL score using the Danish utility weights [19].

The five-level version has been chosen rather than the three-level version, due to its potentially greater sensitivity and reliability, though there are currently no Danish utility weights available for the five-level version [20]. If no country-specific utility weights are available at the planned time of analysis and publication, a crosswalk to the existing Danish EQ-5D-3L utility weights will be performed [21].

The EQ-5D scores and disability scores from each measurement are used to estimate effectiveness, QALYs, and disability over the 1-year period of the clinical trial. QALYs are estimated by multiplying time with the average HRQoL, rendering QALY a composite measure of both quantity and quality of life lived [13].

### Statistical analysis

Analyses will be conducted according to the principle of intention-to-treat in comparisons of the two groups, control and intervention, respectively [22,23]. The economic evaluation will use patient-level data on resource use and effect within the clinical trial period to assess the cost-effectiveness of the extended implementation strategy versus usual implementation.

#### Costs

Cost analyses will include measures of arithmetic means, between-group differences, and variability of differences, as well as testing for statistical significance [22,23]. This will be presented by trial arm, both as a cost per item and as a total cost for each follow-up during the trial period.

#### Effectiveness measures

Effectiveness of the intervention will be analysed in agreement with the CONSORT statement, extended to cluster randomised trials (see Additional file 1) [24]. Effectiveness measures applied in the cost-effectiveness analyses correspond to the clinical end points of the trial. Statistical analyses of these are described in the clinical trial protocol [6,23].

The cost-effectiveness analyses will be completed regardless of whether the clinical trial demonstrates a significant difference in secondary care referrals or any clinically relevant changes in disability, as recommended by Ramsey et al. [23].

Difference in arithmetic mean QALY will be applied as the effectiveness measure in the cost-utility analysis. For the EQ-5D-5L index values, measures of central tendency and dispersion will be presented for both groups at baseline and after 4, 8, and 52 weeks. These will be presented along with median values as well as the 25th and the 75th percentiles, as recommended by the EuroQol Group [19]. Assessment of outcomes will be blinded.

#### Missing data

In health economic evaluations alongside clinical trials, the presence of missing data, other than censoring, is often to be expected [22,23]. However, due to the extensive registration of, among others, activities and referrals in primary care for all Danish citizens in the Danish National Health Insurance Service Register [25], data on referrals to secondary care is expected to be close to complete. Data completeness on patient-related effectiveness measures is expected to be somewhat lower. Multiple imputations will be carried out if missing data is considered to be missing at random or missing completely at random, but not if it is deemed to be missing not at random [23,26,27].

#### Regression analysis

To adjust for baseline covariates, regression analysis of cost and QALY will be applied. Included baseline covariates will be: age, gender, history of low back pain episodes, length of low back pain episode, comorbidity, pain intensity, disability, and HRQoL (for the QALY regression). Generalised linear models are considered because cost, as well as QALY, does not follow a Gaussian distribution [28].

#### Cost-effectiveness

The economic evaluation is conducted as a within-trial analysis, where the cost-effectiveness will be presented in terms of ICERs, calculated as the arithmetic mean difference in cost between the extended implementation strategy and the usual implementation strategy divided by the arithmetic mean difference in effect [13,22].

A number of sensitivity analyses are considered to quantify the level of decision uncertainty, e.g. cost-effectiveness scatterplots and cost-effectiveness acceptability curves. Deterministic sensitivity analyses will be performed on chosen variables to identify key determinants for the results. For instance, it is anticipated that if the pop-up systems, i.e. STarT and SOS, were to be implemented in general practice, the costs of implementation should be amortised over the lifetime of the asset. On these grounds, the impact of amortising the costs will be evaluated in a sensitivity analysis to investigate whether implementation costs might be a key cost driver. Finally, sensitivity analyses are made for complete cases and are unadjusted for baseline covariates.

A 5% significance level is set for all models. STATA/MP 12.1 will be used for statistical analysis.

### Ethics

For the clinical trial, the Regional Scientific Ethics Committee and the Danish Health and Medicines Authority did not find that approval was a necessity. The clinical trial is registered with the Danish Data Protection Agency, the Committee of Multipractice Studies in General Practice, and at ClinicalTrials.gov (NCT01699256). Further detail on compilation of various consent forms for participation in the clinical trial is provided in the protocol for analysis of the effectiveness of the intervention [6].

## Discussion

This paper represents the protocol of an economic evaluation nested in a cluster randomised controlled trial, which aims to assess the cost-effectiveness of an extended implementation strategy compared to usual implementation of low back pain guidelines in general practice. The demand to provide evidence of value for money has resulted in an increased number of economic evaluations performed alongside clinical trials; however, there are some difficulties in the design of these economic evaluations [29]. Following internationally recognised guidelines [23], this protocol serves to heighten the transparency of the economic evaluation.

Due to the setup of a clinical trial, Ramsey et al. and O’Sullivan et al. place great importance on identification of any possible threats, such as recruitment to the study, protocol-driven utilisation, and enhanced compliance, to the external validity of the economic evaluation [23,29]. As recruitment to the study is of importance to the generalisability and transferability, a multicentre approach is adopted in this study to allow for variations across general practices [30], albeit limited to the North Denmark Region.

Protocol-driven utilisation covers consumed resources not normally consumed in standard clinical care and are also often related to compliance, which constitutes a complex issue in this study. This study aims to enhance general practitioners’ adherence to the clinical guidelines by comparing two different strategies of implementation—usual dissemination and extended implementation, respectively—with usual dissemination representing the normal approach to implementation of new guidelines. Compliance attributed to the intervention may be impeded, as participation and continuous focus on a specific area in a clinical trial can induce artificially enhanced compliance. Inevitably, this also affects compliance in the usual care branch and can bias the economic outcomes by possibly overestimating the costs and effects in the clinical trial relative to a real-world setting. This necessitates the need to compare compliance to the clinical guidelines through clinical practice patterns between general practices enrolled in the study and those not participating.

In conclusion, it is expected that the extended implementation strategy will reduce the number of patients referred for secondary care and improve patient-related outcomes. It is hypothesised that the additional upfront cost of extended implementation will be counterbalanced by improvements in clinical practice and patient-related outcomes, thereby rendering the extended implementation strategy cost-effective.

## Additional file

Additional file 1: Table S1. CONSORT 2010 checklist of information to include when reporting a cluster randomised trial.

## Abbreviations

DAK-E: Danish quality unit of general practice
DREAM: The Danish Register of Sickness absence and compensation benefits and social transfer payments
EQ-5D-5L: EuroQol 5-dimension 5-level
HRQoL: Health-related quality of life
ICER: Incremental cost-effectiveness ratio
ICPC: International classification for primary care
Nord-KAP: quality unit for general practice in the North Denmark Region
QALY: Quality-adjusted life-year
RDQ: Roland Morris disability questionnaire
SOS: Social risk screening
STarT: Subgroups for targeted treatment back screening tool
VAS: Visual analogue scale
